# Trajectories of Psychosocial Phenotypes in Older Adults With Pain: A Latent Transition Analysis

**DOI:** 10.1093/geroni/igaf122.3083

**Published:** 2025-12-31

**Authors:** Yu-Ping Chang, Ashleigh Holmes

**Affiliations:** University at Buffalo, Buffalo, New York, United States; New York University, New York, New York, United States

## Abstract

Despite the high prevalence of pain in older adults, research on multivariable contributors to pain outcomes remains limited. Psychosocial phenotyping, which identifies patterns of psychological and social characteristics, requires further longitudinal analysis to better understand its role in older adults experiencing pain. This study explored the stability of psychosocial phenotypes longitudinally in older adults with pain and identified predictors of phenotype transitions over time. Using 2018-2022 annual data from the National Health and Aging Trends Study, latent transition analysis was performed in older adults consistently reporting pain (n = 813) to assess psychosocial phenotype transitions longitudinally. Psychosocial phenotypes comprised clusters of similar scores on depression, anxiety, affect, self-realization, resilience, self-efficacy, and social participation. Baseline variables (pain characteristics, biological, cognitive) associated with transitions in phenotypes were determined via logistic regression. Three psychosocial phenotypes (Adverse, Favorable, and Intermediate) were identified. Longitudinally, phenotype membership remained largely stable with a trend towards increased psychosocial adversity. A temporary decline in psychosocial well-being was observed during the COVID-19 pandemic, highlighting the impact of major stressors on these trajectories. Baseline cognition and physical performance predicted transitions to less adverse phenotypes, whereas poorer sleep, diminished physical performance, greater pain-related limitations, and lower self-rated health predicted transitions toward more adverse phenotypes. Older adults with pain exhibit a notable degree of psychosocial stability and resilience, even in the face of significant external stressors. Future research should explore interventions that promote transitions to more favorable phenotypes, support point-of-care clinical decision-making, and advance the integration of precision medicine approaches in pain management.

